# Primary care mental health services in Qatar

**DOI:** 10.1192/bji.2020.45

**Published:** 2021-02

**Authors:** Ovais Wadoo, Mohamed Ali Siddig Ahmed, Shuja Reagu, Samya Ahmad Al Abdulla, Majid Ali Y. A. Al Abdulla

**Affiliations:** 1 Senior Consultant Psychiatrist, Psychiatry Department, Hamad Medical Corporation, Doha, Qatar. Email: owadoo@gmail.com; 2 Senior Consultant Family Physician, Deputy National Mental Health Lead, and Executive Director of Operation Primary Health Care Corporation, Doha, Qatar; 3 Senior Consultant Psychiatrist, Chairman, Mental Health Services, Hamad Medical Corporation and Qatar University, Doha, Qatar

**Keywords:** Community mental health teams, primary care, psychosocial interventions, patients, out-patient treatment

## Abstract

With rapid growth and development in recent decades, the State of Qatar has been redefining strategies and policies towards building a world-class healthcare system. Mental health has emerged as a priority area for development. As a result, mental health services in the region are being redefined and expanded, and this was realised with the launching of the ambitious National Mental Health Strategy in 2013. Traditionally, mental healthcare in Qatar had been considered to be the remit of psychiatrists within secondary care. The new strategy supported the transition towards community-based care. It outlined a plan to design and build a comprehensive and integrated mental health system, offering treatment in a range of settings. In this article, we provide an overview of the advent of primary care mental health services in Qatar. We discuss the historical aspects of psychiatric care and development of primary care mental health services in Qatar.

Mental health problems are very common globally and constitute a significant proportion of morbidity seen in primary care. Thirty per cent of people seen in primary care have a mental health component to their illness.^1^ There is a high degree of comorbidity between common mental disorders and non-communicable conditions such as cardiovascular disease, diabetes and cancer. A third of people with chronic illnesses such as diabetes have comorbid mental health problems, including anxiety or depression. In addition, many patients in primary care present with physically unexplained symptoms. There is overwhelming evidence in support of delivering integrated physical and mental healthcare.^2^ Primary care is the first level of contact of individuals, the family and the community with the health system, bringing healthcare as close as possible to where people live and work.^3^ The key advantages of delivering mental healthcare through primary care are that it is accessible, acceptable and ensures continuity of care.

## Public health burden of mental disorders in Qatar

Qatar is a peninsula situated halfway down the western coast of the Arabian Gulf, bordered to the south by the Kingdom of Saudi Arabia. Qatar has a population of 2.7 million.^4^ Qatar is one of the world's wealthiest nations in terms of per capita GDP. The prevalence of mental health problems in Qatar is comparable with international data. One-fifth of adults attending primary care have a diagnosable mental disorder. Anxiety and depression are the most common psychiatric presentations in Qatar.^5,6^ The public health burden of mental disorders is high. Anxiety and depression rank fourth in the global burden of diseases. Depression is second only to heart disease as a cause of disability in Qatar.^7^

## History of mental health services in Qatar

The provision of mental health services in Qatar was first established in 1971.^8,9^ Hamad Medical Corporation (HMC) was established in 1982 and is the main public provider of healthcare in the country, including mental health services. For many decades, the mental health service was a traditional hospital-based service provided by mental health specialists. It included in-patient, out-patient and emergency services.^10^ The National Mental Health Strategy for Qatar, *Changing Minds, Changing Lives, 2013–2018*, was launched in 2013.^11^ This was a ground-breaking policy initiative which focused on providing most mental health services in primary and community settings close to the populations served, in line with the best available evidence. The strategy supported the transition towards community-based care. It outlined a plan to design and build a comprehensive and integrated mental health system, offering treatment in a range of settings, including a focus on primary care. This initiative was further supported by the publication of the *National Health Strategy 2018–2022*, which specifically identified mental health and well-being as a priority area of development for the nation.^12^

## Primary care mental health services in Qatar

The origin of primary healthcare dates back to 1954. The Ministry of Health in Qatar gave approval for a comprehensive primary healthcare system in 1978.^13^ There was no formalised primary care mental health service; family physicians received no specific training in mental health, and their role was limited to assessment, diagnosis and prescribing for low-level mental health issues. Despite the introduction of primary care services, the majority of mental health services were provided through secondary care. It was identified that non-specialist physicians in primary healthcare were not well trained in mental health. A survey of physicians found that 69% had no training in mental health, and the default was to refer people on to secondary care; 97% of those with mental health problems were referred to specialist services.^14^ The Primary Health Care Corporation (PHCC) was made an independent body in 2012 and is responsible for delivering primary healthcare to the population of Qatar. In line with the National Mental Health Strategy and supported by the National Primary Care Strategy, the Ministry of Public Health set out a plan to establish the primary care setting as the key player in the treatment of psychiatric disorders, to constitute the basis of the mental health service delivery system and improve access to mental health services. PHCC is state-funded and provides primary healthcare services through an organised network of family physicians and allied health staff at its countrywide health centres.^13,15^ PHCC has established a joint working relationship with HMC to enhance collaboration, with a clear focus on the development of an integrated network of services and agreed pathways among all providers of mental health services. The importance of collaboration between specialised mental health and primary care services to enable primary care to deliver effective mental healthcare is well established.^3^ The provision of formal mental health services was piloted in the primary care setting in December 2014 in three centres soon after the publication of the National Mental Health Strategy. This was followed by provision of psychological therapies in the primary care setting in December 2016. The delivery of integrated care had to address issues of training, continuing education, practice guidelines, supply of psychotropic medication, support, health information systems, information sharing and liaison between primary and specialist care before it was rolled out to all the centres in the country. To support the integration, each health centre was allocated a lead physician as a nominated Mental Health Champion, who acted as the portfolio holder overseeing the process of integration. Preliminary results at the pilot PHCC centres indicate that there has already been significant progress; the primary health centres routinely screen patients for anxiety and depression through the use of screening tools and are now geared to treat common psychiatric disorders. The guidelines specifically developed for integrated working encourage physicians to manage patients using the stepped care approach, with a combination of medication and psychological therapies and referrals of more complex patients to secondary care. More recently, provision of specialist mental health services in primary care settings has also been initiated in some centres. This new service aims to provide accessible community-based specialist care to patients.^16^ In line with the strategy, specialist general adult and geriatric psychiatry clinics have been started in some primary health centres. The clinics are run by psychiatrists in what patients perceive as a less stigmatising setting. This is more acceptable and allows for better and increased identification of mental health issues and optimal management of patients with a psychiatric disorder within the primary care environment. Such clinics are known to improve patient satisfaction and decrease healthcare costs.

## Education, training and support for primary care

A key factor underlying the success of this programme for integration has been continuing education, training and support of PHCC physicians in mental health issues. This is supported by evidence of integrated working from other countries, which highlights that primary care physicians with mental health training see more patients with mental health presentations and treat a higher proportion of them compared with primary care physicians without this training.^17,18^ Family physicians and nurses were provided with foundation mental health training, and some family physicians received advanced mental health training to support the delivery of mental health services in primary care. PHCC commissioned Maudsley International to help train 600 physicians in foundation mental health; 60 of these physicians received advanced mental health training, and 1800 nurses also received mental health awareness training from mental health clinical leads within PHCC to manage patients presenting with mental health symptoms. The training was focused on mild-to-moderate mental health issues, namely depression and anxiety. This training and support started before the roll-out of the programme of integrated working.^9^ PHCC has an ongoing training programme in mental health for its workforce. As part of collaborative efforts to improve delivery of primary care mental health services and, more specifically, the primary–secondary care interface, specialists from HMC have initiated a training programme to promote mental health education and training for primary care physicians. This includes continuing education workshops and presentations that are relevant to the realities and demands of primary care and the interface with secondary care.

## Challenges and recommendations

Family physicians in Qatar come from diverse backgrounds and have varying levels of training and exposure to treating mental disorders; some family physicians may perceive insufficient knowledge or experience as a barrier to management of mental disorders. Furthermore, some family physicians have limited motivation to manage mental health problems. It is important to create learning and support opportunities for less connected family physicians or those primary care personnel who have lacked interest in or conviction about empowering mental health at the primary care level. Mental health champions have an important role in identifying and supporting such physicians. There is a licensing requirement by the regulatory body for primary care physicians to maintain continuous professional development (CPD), but this does not include mandatory mental health credits. The authors are of the opinion that primary care physicians should be able to evidence learning in mental health as part of their CPD, assessed annually at their appraisal, and should undertake regular suicide prevention training in the same way that they are expected to undertake life support training. It is important that not only current primary care professionals but also trainees who represent the future workforce have access to appropriate training and education to meet the needs of psychiatric care provision. The Family Medicine Residency Program in Qatar is a 4-year programme fulfilling the requirements of the Accreditation Council of Graduate Medical Education for the accreditation process. The residency training programme for family physicians includes psychiatry placements. The authors are of the opinion that this programme could be extended and focused on developing primary psychological skills. Psychological interventions are at the core of primary care psychiatry. The need to recruit and adequately train psychologists who are front-line staff to provide psychological therapies remains a challenge. Qatar has a high proportion of single male migrant labourers. The primary care provider for this population is Qatar Red Crescent (QRC). At present, QRC is not geared to treat common psychiatric disorders. Psychiatric care of such patients is currently limited to secondary care services.

## Conclusions

Primary healthcare is increasingly recognised as an integral part of Qatar's mental healthcare structure and delivery. Qatar has developed a comprehensive primary healthcare system and continues to expand its facilities and workforce. These changes have been underpinned by a clear mental health strategy, and present Qatar as a model for other countries in the Arabian Gulf region when developing and enhancing their own primary care mental health services.

## Supplementary material

For supplementary material accompanying this paper visit https://doi.org/10.1192/bji.2020.45. click here to view supplementary material
